# Assessment of immune functions and MRI disease activity in relapsing-remitting multiple sclerosis patients switching from natalizumab to fingolimod (ToFingo-Successor)

**DOI:** 10.1186/s12883-015-0354-9

**Published:** 2015-06-23

**Authors:** Luisa Klotz, Berit Grützke, Maria Eveslage, Michael Deppe, Catharina C. Gross, Lucienne Kirstein, Anita Posevitz-Fejfar, Tilman Schneider-Hohendorf, Nicholas Schwab, Sven G. Meuth, Heinz Wiendl

**Affiliations:** Department of neurology, University Hospital Münster, Albert-Schweitzer-Campus 1, building A1, Münster, 48149 Germany; Institute of biostatistics and clinical research, Westfaelische Wilhelms-University Münster, Münster, Germany

**Keywords:** Multiple sclerosis, Natalizumab, Fingolimod, PML, Switching

## Abstract

**Background:**

In light of the increased risk of progressive multifocal encephalopathy (PML) development under long-term treatment with the monoclonal antibody natalizumab which is approved for treatment of active relapsing remitting multiple sclerosis (RRMS), there is a clear need for alternative treatment options with comparable efficacy and reduced PML risk. One such option is fingolimod, a functional sphingosin-1-receptor antagonist that has been approved as first oral drug for treatment of active RRMS. However, the optimal switching design in terms of prevention of disease reoccurrence is still unknown. Moreover, potential additive effects of both drugs on immune functions, especially with regard to migration, have not yet been evaluated.

**Methods/design:**

This is an exploratory, open-label, monocentric, investigator-initiated clinical trial. Fifteen RRMS patients under stable treatment with natalizumab will receive one last natalizumab infusion followed by a wash-out period of 8 weeks before fingolimod treatment initiation for a period of 24 weeks. Disease activity under natalizumab and during switching will be closely monitored by assessment of relapse rate and disease severity as well as high-frequent high-resolution magnetic resonance imaging including quantitative diffusion tensor imaging. Immunological assays include longitudinal assessment of adhesion molecule expression, functional evaluation of the migratory capacity of immune cells in an in-vitro model of the blood–brain-barrier, and the quality of cellular antiviral immune responses.

**Discussion:**

Our trial represents the first detailed and longitudinal functional analysis of key immunological parameters in the process of switching from natalizumab and fingolimod, especially with regard to potential additive effects of both drugs on trafficking and immune surveillance. Moreover, our study will generate valuable information about even subtle disease exacerbations as consequence of natalizumab cessation, which will help to understand whether a switching protocol containing a wash-out period of 8 weeks before fingolimod treatment is appropriate in terms of disease stability.

## Background

Multiple sclerosis is a chronic autoimmune disease of the central nervous system (CNS) characterized by invasion of inflammatory immune cells into the CNS accompanied by destruction of myelin and axons [1]. In most cases, the disease initially takes a relapsing-remitting course with acute relapses followed by complete or partial recovery. This relapsing-remitting disease course is particularly responsive towards currently available immunomodulatory therapies [1].

One such compound is natalizumab, a recombinant humanized monoclonal antibody that binds to the α4-subunit of α4β1 and α4β7 integrins [2]. Natalizumab effectively interferes with immune cell transmigration across the endothelial cell layer at the blood–brain-barrier (BBB) [3]. This mechanism of action has proven very successful, as clinical studies documented a profound reduction in the annual relapse rate of 68 % and a 42 % reduction in disability progression compared to placebo [2]. However, long-term application of natalizumab has been limited by a rare but serious complication, i.e. progressive multifocal leucoencephalopathy (PML), an opportunistic brain infection caused by JC virus reactivation. The underlying mechanism of natalizumab-associated PML is still unknown, but it has been demonstrated that disease incidence increases with treatment duration: For JCV positive individuals (50-60 % of the general population) after 2 years of therapy, risk increases to 1:400, which is further increasing to ≈ 1:100 in case of previous immunosuppressive therapy [4, 5]. Hence, this indicates that high efficacy correlates with compromised immune surveillance and increased PML risk.

In the last years, new assays have been developed to improve PML risk estimation during natalizumab treatment [6, 7], but so far, it is still challenging to safely identify pre-PML-cases and prevent disease onset by prompt treatment interruption and natalizumab elimination procedures. Therefore, with increasing PML risk individual future treatment options have to be re-considered and hence a proportion of patients will opt to discontinue natalizumab treatment. However, several studies documented a profound increase in disease activity upon natalizumab discontinuation. In these patients, clinical and MRI disease activity remained low for the first 3 months and increased by the 4th month following dose cessation [8–10]. This particular temporal pattern suggests that recurrence of disease activity follows pharamacokinetics of natalizumab elimination and receptor saturation [11, 12].

The time-course of reconstitution of immune functions upon natalizumab cessation however is completely unclear, and hence, a sound recommendation with regard to a normalized PML risk after treatment discontinuation cannot be given based on the data available so far. Although a direct link to the increased PML risk has to be proven, a study by Stüve and colleagues demonstrated an altered CD4+/CD8+ T-cell ratio in the cerebrospinal fluid (CSF) of natalizumab patients, which was normalized after 6 months of treatment cessation [13]. Moreover, data from our group suggest that certain immune functions especially with regard to the migratory activity of immune cells might be fundamentally altered under long-term natalizumab treatment as for example reflected by increased PSGL-1 expression [14], suggesting that a short wash-out period might not be sufficient for regaining full immune competence.

For patients that had been treated with natalizumab, disease-modifying treatment has to be re-initiated to prevent disease exacerbation. Due to the fact that natalizumab is approved for patients who did not respond properly to first-line therapies such as Interferon-beta (IFNβ) or glatirameracetate (GA) or exhibited high pre-treatment disease activity, there is a clear need to continue treatment with an appropriate immunomodulatory agent such as fingolimod.

Fingolimod (FTY720) - Gilenya® is an oral immunomodulatory drug that serves as a functional antagonist of the sphinosine-1-phosphate (S1P) receptor family resulting in lymphocyte sequestration within lymph nodes [15, 16]. In two large clinical studies (TRANSFORMS and FREEDOMS), fingolimod showed a significant reduction of the annual relapse rate compared to either placebo or the active comparator IFNβ-1a and also significantly improved MRI findings [17, 18]. Mechanistically, immune cell sequestration predominantly affects central memory T-cells (TCM) and naïve T-cells (Tn), whereas effector memory T-cells (TEM), which are critically involved in immune surveillance are relatively spared [19]. It is not clear whether these differential effects on immune cell trafficking might also impair effective antiviral immunity during systemic infections, either against newly acquired or persisting latent viruses. In the clinical studies, two fatal cases of herpes-virus infections occurred under fingolimod treatment, and hence, sufficient immunity against varizella-zoster-virus (VZV) has to be documented before fingolimod treatment initiation. One study by Ricklin *et al.* observed an increased incidence of latent VZV reactivation in saliva of fingolimod-treated MS patients [20]. In a recent analysis of post-marketing data, an increased incidence of VZV-reactivation compared to other immune-modulatory drugs has been described, however, an increased incidence of severe or atypical herpes virus infections has not been observed [21]. So far, only one case of natalizumab-independent PML has been described under fingolimod; however, several PML-cases occurred after switching from natalizumab to fingolimod and were regarded as “carry-over-PML”[22–24].

Due to the fact that both drugs interfere with immune cell trafficking albeit via different mechanisms, it is crucial to understand the distinct effects of these drugs on immune cell trafficking and immune reconstitution in the clinical relevant situation of switching immune-modulatory treatment from natalizumab to fingolimod.

The goal of our open, prospective, monocentric trial in patients with RRMS is therefore to improve our understanding of drug-induced changes in immune cell migration and immune cell function and its consequences on anti-viral immunity and to monitor its impact on clinical and paraclinical disease activity in RRMS patients where natalizumab treatment will be replaced by fingolimod treatment.

## Methods/design

### Trial design

ToFingo-successor is a 32-week, open, monocentric, prospective, exploratory, single-arm study that is conducted at the Department of Neurology, University hospital Münster. Recruitment has been started in April 2014. Fifteen patients with RRMS (according to the 2010 revised McDonald criteria) [25], who are currently treated with natalizumab for at least 12 months and where treatment discontinuation is considered for at least one of the following reasons can be included in this study: treatment duration for more than 2 years, positive JCV antibody status, adverse effects including hypersensitivity reactions, presence of anti-natalizumab neutralizing antibodies, or any other valid medical reason. One last natalizumab infusion is part of the trial followed by a wash-out period of 8 weeks before treatment initiation with fingolimod. During the study, patients are regularly monitored by clinical and MRI assessments to detect early signs of disease reactivation. Blood sampling to collect peripheral immune cells for subsequent immunological assessments is done every 4 weeks, moreover, sequential CSF analysis is an optional part of the study protocol.

The study is approved by the local ethics committee and the German competent authority (Federal Institute for Drugs and Medical Devices). The trial is registered at Clinicaltrials.gov (NCT02325440) and will be conducted in accordance with the Declaration of Helsinki and the guidelines of the International Conference on Harmonization of Good Clinical Practice (ICH-GCP) as well as the applicable German laws. All participants will be required to give written informed consent. Monitoring will be performed according to ICH-GCP.

### Participants

The inclusion criteria for participation included diagnosis of definite RRMS according to the revised 2010 McDonald criteria [25], an age of 18 to 65 years, a score of 0–6.0 on the expanded disability status scale (EDSS) [26], and current immunomodulatory treatment with natalizumab for at least 12 months where treatment discontinuation is considered for at least one of the following reasons: treatment duration for more than 2 years, positive JCV antibody status, adverse effects including hypersensitivity reactions, presence of anti-natalizumab neutralizing antibodies, or any other valid medical reason (Table 1).

**Table 1:** Eligibility criteria

**Inclusion criteria**	Written informed consent provided
	Male and female subjects aged 18 to 65 years
	Diagnosis of RRMS, defined by revised McDonald criteria (2010)
	Expanded Disability Status Scale (EDSS) at screening score ≤ 6.0
	Treatment with natalizumab for at least 12 months prior to screening and consideration of treatment discontinuation for any of the following reasons:
	• treatment duration > 2 years
	• positive JCV antibody status
	• adverse effects including hypersensitivity reactions
	• presence of anti-natalizumab neutralizing antibodies
	• any other valid medical reason
**Exclusion criteria**	History of chronic disease of the immune system other than MS, which requires systemic immunosuppressive treatment, or a known immunodeficiency syndrome.
	Diagnosis of Crohn’s disease or ulcerative colitis
	Treatment with:
	• systemic corticosteroids or immunoglobulins within 1 month prior to baseline
	• immunosuppressive medications such as azathioprine, cyclophosphamide or methotrexate within 3 months prior to baseline
	• monoclonal antibodies (excluding natalizumab) within 3 months prior to baseline.
	• cladribine or mitoxantrone at any time.
	History of malignancy of any organ system (other than cutaneous basal cell carcinoma)
	Uncontrolled diabetes mellitus (HbA1c >7 %)
	Diagnosis of macular edema during Screening Phase
	Severe active infections, active chronic infection.
	Negative for varicella-zoster virus IgG antibodies prior to baseline.
	Vaccination with live or live-attenuated vaccine (including varicellazoster virus or measles) within 1 month prior to baseline
	Previous therapy with total lymphoid irradiation or bone marrow transplantation
	Any medically unstable condition, as assessed by the investigator
	Any of the following cardiovascular conditions and/or findings in the screening ECG:
	• history of cardiac arrest
	• myocardial infarction within the past 6 months prior to screening or with current unstable ischemic heart disease
	• history of angina pectoris due to coronary spasm
	• heart failure (NYHA III-IV) or any severe cardiac disease as determined by the investigator
	• history or presence of a second-degree AV block, Type II or a third-degree AV block or QTc interval > 450 ms in males and > 470 ms in females
	• treatment with Class Ia (ajmaline, disopyramide, procainamide, quinidine) or Class III antiarrhythmic drugs (e.g., amiodarone, bretylium, sotalol, ibulitide, azimilide, dofelitide)
	• proven history of sick sinus syndrome or sino-atrial heart block
	• uncontrolled hypertension
	• Resting HR lower than 45 bpm
	Presence of severe respiratory disease, pulmonary fibrosis or chronic obstructive pulmonary disease (Class III-IV)
	Any of the following hepatic conditions:
	• severe hepatic injury (Child-Pugh class C)
	• total bilirubin greater than 2 times the upper limit of the normal range
	• AST or ALT greater than 2 times the upper limit of the normal range
	• alkaline phosphatase (AP) greater than 2 times the upper limit of the normal range
	White blood cell (WBC) count <3,500/mm^3^ or
	lymphocyte count <800/mm^3^ at screening
	Any of the following neurologic/psychiatric disorders:
	• history of substance abuse (drug or alcohol) in the past five years or any other factor (i.e., serious psychiatric condition) that may interfere with the subject’s ability to cooperate and comply with the study procedures
	• progressive neurological disorder, other than MS, which may affect participation in the study
	Incapability to undergo MRI scans, including claustrophobia or history of hypersensitivity to gadolinium-DTPA
	Treatment with investigational drug or therapy within 180 days or 5 half-lives before baseline, whichever is longer
	Pregnancy or breast feeding (lactating), confirmed by a positive hCG laboratory
	Child-bearing potential (Unless the women concerned are using effective contraception during the study and for 5 half-lives after stopping treatment. In case of use of oral contraception women should have been stable on the same medication for a minimum of 3 months before baseline)
	History of hypersensitivity to the study drugs or to drugs of similar chemical classes
	Prior participation in a trial with fingolimod

The main exclusion criteria include any disease course other than RRMS, recent immunosuppressive treatment, a history of malignancy (except cutaneous basal cell carcinoma), severe active infections, patients negative for VZV IgG antibodies prior to baseline, medical conditions prohibiting treatment with fingolimod (such as severe diabetes mellitus, relevant cardiovascular conditions, severe respiratory disease or severe hepatic injury), relevant white blood cell abnormalities, patients unable to undergo MRI scans, and pregnant or lactating women. The full list of exclusion criteria is depicted in Table 1.

### Interventions

Patients’ eligibility will be determined during screening. At the baseline visit, patients will receive their last natalizumab infusion. Optionally, CSF analysis will be performed at this time point to detect natalizumab-induced changes in immune cell subset composition in the CSF. MRI imaging will be performed to determine disease activity under natalizumab treatment and to screen for possible PML-induced changes. During the defined 8 week wash-out phase, patients will be clinically monitored every 4 weeks. After wash-out, fingolimod treatment will be started and visits will be carried out every 4 weeks for the next 24 weeks of fingolimod treatment. MRI scans will be performed at the time of fingolimod initiation and at weeks 4, 8, 16, and 20 after treatment initiation, respectively. Optionally, a second CSF analysis will be performed 24 weeks after initiation of fingolimod treatment. Optional follow-up visits will be offered at week 28 and week 32 after fingolimod treatment initiation. Blood sampling for subsequent immunological analyses will be performed at each visit. If necessary, unscheduled visits can be performed at any time. Table 2 illustrates the timeline of all interventions. An overview of the immunological asays is provided in Table 3.

**Table 2:** Schedule of regular study visits

Study Phase	Screening	Wash-out			← Treatment →				Follow up			
Visit*	**1**	**2**	**3**	**4**	**5**	**6**	**7**	**8**	**9**	10**	**11**	**12**
**Study Week**	**−4 – 0**	**0**	**4**	**8**	**12**	**16**	**20**	**24**	**28**	**32**	**36**	**40**
Obtain informed consent	X											
Background/Demography	X											
Inclusion/exclusion criteria	X	X										
Medical History	X											
MS history/MS treatment	X											
Prior/Concomitant medication	X	X	X	X	X	X	X	X	X	X	X	X
Pregnancy test (serum)	X											
Pregnancy test (urine dipstick)		X	X	X	X	X	X	X	X	X	X	X
Physical examination	X	X	X	X	X	X	X	X	X	X		
MSFC	X^6^	X								X		
Neurological examination (including EDSS)	X	X	X	X	X	X	X	X	X	X		
Vital signs	X	X	X	X	X	X	X	X	X	X	X	X
Clinical laboratory (blood chemistry/hematology)	X	X	X	X	X	X	X	X	X	X	X	X
PBMC isolation for evaluation of immunological parameters		X	X	X	X	X	X	X	X	X	X	X
CSF sampling^1^		X								X		
JCV sample	X									X		
Ophtalmologic examination^2^	X						X					
Last natalizumab dose		X										
ECG	X											
First dose monitoring (=FDM)^3^				X								
Dispensing of patient’s diary				X								
Collecting of patient’s diary										X		
Study drug prescription				X	X			X				
MRI (including DTI)^4,5^		X		X	X	X		X		X		
MS relapse	X	X	X	X	X	X	X	X	X	X	X	X
Adverse events/SAEs		X	X	X	X	X	X	X	X	X	X	X

*At each visit the patient must be reminded of the importance of remaining vigilant for infections, performing regular skin selfassessments, and in women of child bearing age, practicing contraception per protocol

** EOT/ Assessments also for PPW (Premature Patient Withdrawal)

^1^In several patients (estimated number: 8–10), CSF specimen will be obtained under long-term natalizumab (i.e. baseline) and

after 24 weeks of fingolimod therapy and will immediately be analyzed by flow cytometry

^2^Patients with a history of uveitis or with diabetes mellitus must perform an ophthalmologic examination prior to the treatment start of fingolimod. Ophthalmologic examination includes eye history and procedures necessary to assess macular edema (e.g. ophthalmoscopy). OCT is only done to confirm macular edema

^3^Patients will be monitored on-site in the clinic for at least six hours after dose administration at First Dose Monitoring (FDM) visits (see Appendix 3 for details). Pulse and blood pressure will be obtained three times for pre-dose assessment. The monitoring includes a 12-lead ECG prior and 6 hours after the first dose, as well as blood pressure and heart rate measurement every hour

after the first dose

^4^Window for MRI is +/− 7 days

^5^If a patient discontinues study treatment early due to relapse and last MRI was > 10 days from relapse confirmation, an MRI should be performed, if possible, prior to initiation of corticosteroid treatment

^6^MSFC at Screening (V1) should be taken as rehearsal

**Table 3:** Immunological Assays

	Biomaterial		
Assay	PBMC^1^	PB Immune cells^2^	CSF^2^
Flow cytometric assessment of CD49d and CD62L expression	X		
*In vitro* transmigration across BBB	X		
VZV-specific T-cell response	X		
Immune phenotyping via flow cytometry	X	X	X

^1^purified via density gradient centrifugation and cryo-preserved

^2^freshly obtained, treated with erythrocyte-lysis buffer

### Outcome parameters

#### Primary endpoints

The co-primary endpoints are to investigate the effect of switching therapy from natalizumab to fingolimod on peripheral blood immune cells regarding 1) CD49d expression over time and 2) changes in their migratory capacity in an in-vitro model of the BBB. It has been demonstrated that natalizumab causes an early and pronounced reduction in T-lymphocyte CD49d expression [14] accompanied by an impaired transendothelial migratory capacity [27]. CD49d expression was shown to be an indicator for an appropriate biological response to natalizumab treatment [28], and wash-out of Natalizumab by plasma exchange restores the transendothelial migratory capacity of lymphocytes [29]. Furthermore, CD62L expression is reduced by Natalizumb therapy *per se* and a strong reduction in CD62L is associated with increased risk of developing PML [6]. We therefore aim to assess changes in these molecules over time in the course of switching.

##### PBMC isolation and cryo-preservation

For all immunological assays using peripheral immune cells, peripheral venous blood (90 ml EDTA blood) will be obtained from all subjects included in this study at different time points as indicated above. PBMCs will be isolated from the blood via density gradient centrifugation as described before [30]. Purified PBMC will be aliquoted and cryo-preserved in liquid nitrogen for at least 4 weeks before analysis. After thawing, the CD49d and CD62L expression on CD4+ and CD8+ T cells will be evaluated via flow cytometry using a Navios™ flow cytometer (Beckmann Coulter, Krefeld, Germany).

##### Transmigration assay

To assess the migratory capacity of immune cells across the BBB a transmigration assay will be used as described by our group [14]: A defined number of PBMC will be added on top of a confluent monolayer of primary human brain endothelial cells seeded on transwells with a pore size of 3.0 μm and left to migrate for 6 h at 37 °C into a medium-filled chamber below. Migrated immune cells will be characterized and quantified via flow cytometry. By correlating cell counts and composition of immune-cell populations before and after the transmigration, potential changes in the migratory capacity of each immune-cell type throughout the study will be evaluated.

##### Secondary endpoints

The secondary endpoints comprise changes in paraclinical disease activity over time upon natalizumab cessation and switching to fingolimod treatment. MRI disease activity is defined as change in T2w lesion load, detection of Gd + lesions as well as changes in T1w and FLAIR lesions compared to the baseline MRI.

MRI acquisitions will be performed with a 3 T MRI scanner (TIM Trio, Siemens AG, Erlangen, Germany) and a 12-channel (matrix) head coil (Siemens AG, Erlangen, Germany). Images of the following MRI sequences will be obtained: a native isotropic 3D MPRAGE T1-weighted sequence; diffusion-weighted echo planar imaging for (DTI); axial turbo spin-echo (TSE) FLAIR; sagittal TSE FLAIR; 3D MPRAGE T1-weighted after intravenous gadolinium-DTPA (diethylene triamine penta-acetic acid) 0.1 mmol/kg injection; axial PD/T2-weighted TSE.

##### Exploratory endpoints

In addition, several exploratory endpoints have been defined. Exploratory immunological objectives comprise the analysis of the functionality of virus-specific T-cell responses and of changes in immune cell subset composition in the course of our study.

Additionally, quantitative DTI imaging will be performed throughout the study in order to search for radiological signs of disease exacerbation that escape detection by conventional radiological methods.

Finally, clinical parameters monitored throughout the whole study shall be correlated with radiological and immunological parameters.

##### Assessment of anti-viral immune responses

To assess the quality of virus-specific cellular responses of CD4+ and CD8+ cells throughout the study and in comparison with immune cells derived from healthy controls and treatment-naïve RRMS patients an established protocol will be followed [31, 32]. In detail, thawed PBMC will be restimulated with VZV antigen. After 5 h at 37 °C, VZV-specific production of TNFa, IFNg and IL-2 by CD4+ and CD8+ memory T cells will be measured via intracellular flow cytometry. This analysis will reveal whether treatment with natalizumab itself or switching to fingolimod might affect cellular immune responses against herpes viruses.

##### Immune cell phenotyping

Changes in immune-cell subset composition and immune-cell phenotype will be evaluated by multi-color flow cytometry; here, we will address effects on T helper cell subsets (i.e. Th1, Th17, Treg subpopulations) CD8 T cells, B cell subpopulations, monocytes, dendritic cells, and NK cells. Moreover, the influence on CD62L expression will be determined. The aim of this exploratory objective is to search for potential immunological alterations in the course of treatment switching, which might affect the quality of the immune response and hence indicate an impairment of cellular immune functions e.g. during infections.

##### CSF analysis

In some participants, CSF will be analyzed at baseline (i.e. under natalizumab treatment) and after 24 weeks of fingolimod treatment. Immune cell subset composition within the CSF will be assessed. Due to the limited amount of material, we will focus on CD4+ and CD8+ T cells, B cells, NK cells and Th17 cells. This analysis aims to determine changes in the immune surveillance capacity in the course of switching from natalizumab to fingolimod.

##### DTI imaging

With regard to radiological disease activity, all patients will be longitudinally screened for “silent” disease activity as detected by quantitative DTI imaging in order to search for radiological signs of disease exacerbation related to natalizumab treatment cessation that escapes detection by conventional radiological read outs. Quantitative DTI parameters (mean diffusivity, radial diffusivity, axial diffusivity, linear diffusivity, planar diffusivity, and spherical diffusivity in selected regions of interest) will be compared longitudinally and with data from an age and gender matched normal control cohort of 30 healthy control subjects.

##### Clinical parameters

Clinical parameters that will be determined during the whole study are number of relapses, defined as new development, aggravation or re-occurrence of a neurological abnormality compatible with MS that lasts for at least 24 h, does not occur in the context of fever or infections and that is separated from a preceding clinical event by at least 30 days. Moreover, clinical disease severity will be assessed by EDSS [26] at each visit. These data serve as parameters for determination of clinical treatment responses and changes in clinical disease activity during the trial. Although due to the limited number of participants these parameters do not represent endpoints on their own, they will be used for correlation with immunological as well as radiological parameters in order to investigate a potential relationship between clinical response and immunological alterations.

### Sample size

The sample size calculation is based on the two primary endpoints CD49d expression over time and changes in the migratory capacity in an in-vitro model of the BBB.

A change of 16 units with a standard deviation of 20 units in CD49d expression from baseline to the end of the washout period is deemed clinically relevant. A number of 15 patients results in a power of 82 % for a two-sided Student’s *t*-test for matched pairs with a significance level of 5 %. A sample size of 15 patients also enables to detect a change in the migratory capacity from baseline to the end of the washout period of 4.6 % with a standard deviation of 5.9 % using a power of 80 % with a two-sided Student’s *t*-test for matched pairs with a significance level of 5 %.

### Statistical methods

Statistical analyses will be performed according to the principles of the ICH guideline E9 using standard statistical software. Endpoints will be evaluated for the full analysis set as well as for the per protocol set that contains patients that were treated at least once with fingolimod and have valid data for at least one visit during the two year treatment phase. Data on safety will be analyzed descriptively including all patients that received at least one dose of the investigational drugs (safety population).

The two primary endpoints will be tested hierarchically using the per-protocol set and student’s *t*-test for matched pairs. Other endpoints will be analyzed descriptively using summary measures such as mean and standard deviation, median and quartiles, or frequency and percent, as appropriate. To assess the difference of measurements between two different time points, statistical tests appropriate to the statistical distribution of the particular endpoint will be used (Student’s *t*-test for paired samples, Wilcoxon signed-rank test, Sign-Test). For comparison of more than two successive measurements, methods accounting for intra-individual correlation will be used (e.g. Linear Mixed Models). Since the study is regarded as an exploratory study (hypotheses generating, not confirmatory), p-values will be interpreted accordingly.

A comparison with two adequate historical control groups (healthy volunteers, n = 20, treatment naïve MS patients, n = 20), for which data has been collected in previous studies, will be carried out descriptively.

## Discussion

The risk of developing PML as a consequence of established treatment with natalizumab restricts its long-term use despite high efficacy. However, natalizumab treatment interruption has been shown to be associated with an increased risk of disease exacerbation [9]. Hence, there is a clear need of treatment regimes that allow for satisfactory disease control after natalizumab treatment cessation without an increased risk of severe side effects due to compromised immune surveillance, such as PML and other infections. Based on the available efficacy data, one compound that seems suitable for treatment of active RRMS after natalizumab discontinuation is fingolimod. Hence, it is important to understand the consequences of switching immune-modulatory treatment from natalizumab to fingolimod both in terms of disease control and in terms of consequences for cellular immune responses.

Several studies have already been dealing with the identification of an optimal wash-out interval before post-natalizumab treatment initiation: In this regard, it is now believed that a wash-out interval beyond 12 weeks will result in a rebound of disease activity; therefore, such a long treatment interruption is not recommended [33–35]. This is also supported by data from a multi-center double-blinded clinical trial performed by Novartis (ToFingo), where three cohorts of patients were switched from natalizumab to fingolimod after a wash-out period of either 8 weeks, 12 weeks, or 16 weeks and disease activity was subsequently monitored. Although this study was prematurely stopped, the data available on 142 patients demonstrate that the 8-week wash-out group exhibited the least amount of clinical disease activity and the smallest change in T2 volume throughout the study (presented at ECTRIMS 2013, abstracts 167 and 179).

On the other hand, a limited wash-out period might be associated with safety issues due to potential synergistic effects of both drugs on immune functions which might hamper immune responses e.g. in the context of infections. After 4 weeks of natalizumab treatment cessation, antibody concentration in serum is still detectable [36] and even 8 weeks after stopping treatment with natalizumab, saturation on the surface of circulating immune cells is 70-79 % (on CD4+ T cells) [12], a value which is hardly lower than the expected 80 % during continuous therapy [37]. Saturation only begins to drop significantly after 10 or more weeks. Therefore, fingolimod treatment initiation after only 4 weeks would result in sustained parallel exposure to natalizumab and fingolimod and we decided on a compromise between clinical efficacy and biological response: a wash-out period of 8 weeks.

Concerning the study duration, we decided to monitor patients for 6 months under fingolimod treatment, as it has been suggested that a potential rebound of disease activity upon switching will occur most likely 1–3 months after fingolimod treatment onset. A follow-up of 6 months after fingolimod treatment initiation is therefore regarded as sufficient to detect potential disease exacerbation due to switching.

Potential additive effects of natalizumab and fingolimod on immune function have not been evaluated so far. With regard to natalizumab, it is interesting to note that the overall immune response is not affected even under long-term exposure, and the risk of general infections is not enhanced. In contrast, there is a selectively increased risk for PML, which is caused by reactivation of a latent JC virus infection [38]. The underlying reason for this selective deficit in immune surveillance with regard to JC-virus under long-term natalizumab is still unsolved. One possible explanation is that immune surveillance of the CNS is impaired due to an unselective interference with lymphocyte trafficking at the blood–brain-barrier. Hence, latent local infections cannot be sufficiently controlled any more. In favor of this hypothesis, it has been demonstrated that the CD4+/CD8+ T-cell ratio is selectively reduced in the cerebrospinal fluid (CSF) but in not the peripheral blood of natalizumab patients (i.e. 0,5 under natalizumab vs 3,7 in untreated MS patients [13]. Interestingly, a similarly reduced CD4+/CD8+ T-cell ratio has also been described in the CSF of HIV-infected patients (i.e. ratio of 0.5), and the risk for an opportunistic PML in HIV infected individuals is a well-known complication of chronic HIV-infection. Moreover, PML has been observed in the context of other immune-suppressive conditions such as idiopathic lymphopenia, hematologic malignancies, after bone-marrow transplantation and upon use of different biologicals such as efalizumab and alemtuzumab, thus linking PML occurrence to either CNS-local (i.e. under natalizumab) or systemic defects in immune surveillance [38].

So far, only one case of PML in a fingolimod–treated MS patient who did not receive natalizumab before has been reported (Novartis). With regard to now approximately 172.500 patient-years of exposure, so far there is no increased risk of PML under fingolimod. However, it has to be taken into account that a few cases of PML under fingolimod after switching from natalizumab have been described; so far, it is not clear whether this is due to “carry-over” PML, i.e. asymptomatic PML, which already started under natalizumab, or whether the gradual use of fingolimod after natalizumab has an impact on disease occurrence [22–24]. Moreover, fingolimod seems to harbor an increased risk for herpes virus reactivations, and a few cases of severe and atypical herpes virus infections have been described so far [21].

In light of these findings, we here present a study design that will contribute to our understanding of changes in disease severity and alterations in immune functions upon switching from natalizumab to fingolimod in patients suffering from active relapsing-remitting multiple sclerosis. In contrast to most of the published reports on clinical outcomes of patient cohorts upon switching, where quality of data analysis is limited due to the observational nature of data collection and the fact that the wash-out period was not standardized, we decided for a highly controlled study design that will generate high-quality data to allow proper assessment of disease activity in terms of MRI-related changes as well as clinical relapse rate in our cohort. In light of the available information regarding the wash-out interval, we decided to focus on an interval of 8 weeks for all patients rather than comparing different wash-out intervals. Concerning the high frequency of patient visits implemented in the study protocol, we aim to collect high-frequent MRI data as well as clinical information allowing for a detailed analysis of subtle changes in disease activity over time in each individual.

A detailed and high-frequent analysis of immune functions over time as implemented in our protocol has not yet been performed before in a comparable patient cohort. We believe that it is pivotal to increase our understanding of potential additive effects of these two drugs on immune surveillance, particular with regard to immune cell trafficking and cellular anti-viral immune responses. Based on our previous experience with monitoring of immune functions under long-term immune-modulatory treatment with natalizumab and fingolimod [6, 39], we designed several assays to evaluate changes in immune functions over time during treatment switch. In particular, we will here focus on changes in the expression of molecules involved in immune cell adhesion and migration, i.e. CD49d and CD62L, as well as changes in the migratory capacity of peripheral immune cells, representing the two co-primary endpoints of our study. Reduced CD49d expression is a well-known effect of natalizumab treatment, and it has already been proposed that high-frequent measurement of CD49d levels in patients over time might help for individual dose adjustments [12]. Hence, restored CD49d expression on peripheral immune cells might serve a reliable parameter indicating disappearance of natalizumab-related pharmacodynamic changes. On the other hand, long-term natalizumab treatment alters CD62L levels on peripheral immune cells, and further decreased CD62L levels on immune cells from long-term natalizumab-treated patients have been identified as potential risk factor for developing PML later on [6]. We therefore want to assess potential changes in CD62L expression over time during switching as this has not been evaluated before. Given the fact that both drugs interfere with immune cell trafficking, and distinct effects on immune cell subset migration across primary human brain endothelial cells by both drugs have been described [27, 39], we want to assess whether we might observe any changes in the migratory capacity of different immune cell subpopulations during switching. It is conceivable that despite a wash-out interval of 8 weeks there still might occur additive effects of both drugs on immune cell trafficking that might indicate potential safety issues with regard to local immune surveillance within the CNS. Moreover, we will perform CSF analysis by flow cytometry in individual study participants first at the end of natalizumab treatment and after 24 weeks of fingolimod treatment in order to assess potential changes in the CD4+/CD8+ T-cell ratio and immune cell activation status (i.e. level of HLA-DR expression on immune cells). We will assess whether potential natalizumab-induced changes in the CD4+/CD8+ T-cell ratio might recover upon switching to fingolimod.

Finally, we will evaluate functional changes in cellular anti-viral responses against the herpes viruses CMV and VZV over time during switching. Recent observational data report an increased risk of herpes virus reactivation under fingolimod [20, 21]. It is unclear, however, whether preceding treatment with natalizumab might further impact cellular immune responses against these viruses.

We are fully aware that the limited patient number will prevent any definitive statements with regard to the efficacy of fingolimod therapy after long-term natalizumab treatment; however, this is not the scope of our study. Instead we want to provide an in-depth analysis of MRI data focusing on clinically silent disease activity in order to better understand the kinetics of potential disease exacerbation during switching and hence allow for subsequent re-adaptation of the current treatment paradigm especially with regard to the wash-out interval later on. Moreover, we want to provide a data set that allows for detection of changes in immune functions during switching in order to further understand the pharmacodynamic effects of both drugs and their successive usage on key immune functions such as migration, immune cell activation and cellular immune responses against viruses.

### Trial status

Recruitment ongoing.
